# Overlooked Mechanisms in Type 1 Diabetes Etiology: How Unique Costimulatory Molecules Contribute to Diabetogenesis

**DOI:** 10.3389/fendo.2017.00208

**Published:** 2017-08-23

**Authors:** David H. Wagner

**Affiliations:** ^1^The Program in Integrated Immunology, Department of Medicine, Webb-Waring Center, University of Colorado Anschutz Medical Campus, Aurora, CO, United States

**Keywords:** autoimmunity, T cell receptor revision, costimulation, type 1 diabetes, etiology of willful acts

## Abstract

Type 1 Diabetes (T1D) develops when immune cells invade the pancreatic islets resulting in loss of insulin production in beta cells. T cells have been proven to be central players in that process. What is surprising, however, is that classic mechanisms of tolerance cannot explain diabetogenesis; alternate mechanisms must now be considered. T cell receptor (TCR) revision is the process whereby T cells in the periphery alter TCR expression, outside the safety-net of thymic selection pressures. This process results in an expanded T cell repertoire, capable of responding to a universe of pathogens, but limitations are that increased risk for autoimmune disease development occurs. Classic T cell costimulators including the CD28 family have long been thought to be the major drivers for full T cell activation. In actuality, CD28 and its family member counterparts, ICOS and CTLA-4, all drive regulatory responses. Inflammation is driven by CD40, not CD28. CD40 as a costimulus has been largely overlooked. When naïve T cells interact with antigen presenting cell CD154, the major ligand for CD40, is induced. This creates a milieu for T cell (CD40)–T cell (CD154) interaction, leading to inflammation. Finally, defined pathogenic effector cells including TH40 (CD4^+^CD40^+^) cells can express FOXP3 but are not Tregs. The cells loose FOXP3 to become pathogenic effector cells. Each of these mechanisms creates novel options to better understand diabetogenesis and create new therapeutic targets for T1D.

Medical advances in infectious diseases have been extraordinary, completely changing human history. The polio vaccine, small pox vaccine, and measles vaccine among others changed modern medicine. Success with infectious diseases has influenced and created a generalized approach to most medical problems. Unfortunately, using that approach for autoimmune disease has fared much less well. Diseases like type 1 diabetes (T1D) and multiple sclerosis (MS) have seen unpredictable and steady increases in incidence over the last half-century, with only limited treatment options and no cure options on the horizon (1). World-wide incidence of T1D has doubled over the past two decades (2). Examples of how diabetes is expanding can be described in former Soviet-Bloc countries where T1D incidence was virtually unheard of decades ago, but now has increased substantially in all age groups examined. Incidence in adults is now surprisingly high, but the highest increase was seen in children aged 0–4 years (2). Speculation as to why the incidence rate is so drastically expanding focused on the introduction of Western-Style diet, in other words high-fat, high-glucose diets (3). Other parameters, such as genetics, could not account for the increases. Like T1D, MS incidence is increasing (4), as is inflammatory bowel disease (IBD) (5), even the more rare Hashimoto’s Thyroiditis is seeing steady incidence increase (6). Another alarming trend is autoimmune comorbidities. Once thought to be unlikely, different autoimmune diseases now are being diagnosed in the same patient; MS and T1D (5); MS and IBD (7); rheumatoid arthritis and T1D (8); psoriasis and T1D (9); and alopecia areata and T1D (10). This is partially due to improved diagnostic techniques, and due to longer survival of patients with autoimmune conditions.

Each autoimmune disease is disparate in symptoms and effect; nonetheless, they share immunologic mechanistic similarities. The integral components of autoimmune disease like infectious disease involve classic immune reactions. During an immunological event, foreign antigens, including viruses, bacteria, funguses, etc., are processed and presented as antigens that lead to T and other cell type activation. This process collectively creates an inflammatory microenvironment. Macrophages or dendritic cells (DCs) at the infection site take up the invading pathogen that then is processed. The pathogen is internalized by engulfment or receptor-mediated uptake, and associates with proteasomes to create antigen fragments. The order of antigenicity is protein ≫ DNA/RNA > carbohydrate > lipid. There are very few lipid antigens, although CD1-bearing cells are able to present lipids through the CD1 complex to activate T cells (11). Once generated, the antigen associates with MHC/HLA in the Golgi–endoplasmic reticular compartment. MHC + antigen then is exported and maintained on the cell surface. Professional antigen presenting cells (APCs) include B cells, macrophages, and DC that express the class II version of MHC. CD4^+^ lymphocytes interact with professional APC to create localized inflammation. CD8^+^ cells interact with MHC-class I type molecules. Under normal immune conditions, CD8^+^ cells respond to virally infected cells. In autoimmune conditions including T1D and MS, CD8^+^ cells can play a role in pathogenesis (12, 13). T cells that carry a specific and unique T cell receptor (TCR) recognize and respond to a specific antigen creating localized inflammation. Under septic conditions, the initial response is pro-inflammatory including activation of TH1 and/or TH17 type cells (14). Under non-septic, autoimmune conditions, the pro-inflammatory phenotype also occurs (15–18). In both scenarios, an appropriately expressed TCR is required, the problem being that in autoimmune diseases T cells arise that recognize and respond to self-antigens. The thymic microenvironment is responsible for generating mature T cells with appropriate TCRs; this means generating T cells that respond to foreign/invader antigens but do not respond to self-antigens. There are, however, medical conditions where self-antigen response is desirable. Transformed cells that result in cancers need to be targeted, and self-antigen reactive T cells perform that function. Maintaining the fine-line homeostatic balance, however, becomes the tricky part.

The interaction of TCR and MHC-Antigen is a crucial aspect of immune function, never more so than during autoimmunity. Autoaggressive T cells have been predicted to slip through the selective pressures of the thymus (19). Alternatively, it has been shown that autoaggressive TCRs can be generated in the periphery through a process termed TCR revision (20–23), thereby subverting central tolerance. It is possible that peripheral negative selection occurs and may be dysfunctional in T1D and other autoimmune diseases. TCR revision contributing to autoimmune disease development has been discussed in a previous review (24). A defining feature to peripherally generated, autoaggressive T cells is expression of CD40 (20, 21, 24–28). Relative to CD40 expression on T cells is what function(s) does it perform? A surprising outcome was the potential role as a costimulus molecule (29–31). Relative to the accepted two-signal model for T cell activation (32, 33), costimulation can play a critical, even decisive, role during autoimmunity.

## The Role of Costimulation and Auto-Aggression

The necessity of costimulatory molecules for T cell activation was described in the two-signal model by Bretscher (32). Signal 1 is antigen specific, mediated by TCR interaction with an antigen/MHC complex. Signal 2 is antigen independent, mediated by receptor–ligand interactions that occur between the T cell and APC. The TCR/CD3–MHC/Ag complex with the assistance of adhesion and addressin molecules expressed on each cell type act in a velcro-like manner to adhere the cells together during antigen recognition creating what is known as the immunologic synapse (34). Signaling within the synapse is bidirectional with each cell contributing to the others full activation. Therefore, dysfunction involving either cell could result in pathogenesis. From the T cell perspective, Jenkins and Swartz suggested that TCR-mediated signals alone, without appropriate costimulus, results in a permanent non-responsive condition called anergy (35). While stimulatory signals are sent from T cell to APC within the synapse, the type of costimulation toward the T cell often dictates the immunologic outcome.

## Immunoglobulin “Ig” Costimulus

One of the first described T cell costimulatory molecules was CD28, which turned out to be a member of a subfamily of proteins that includes ICOS and CTLA-4. This subfamily was categorized as “Immunoglobulin-family costimulus” due to the biochemical structure of the proteins. CD28 is expressed on most naïve, activated, and memory T cells; ICOS and CTLA-4 are expressed on activated T cells and subsets of memory cells (36). CD28 and CTLA-4 interact with CD80 (B71) and CD86 (B72) found primarily on professional APCs. ICOS interacts only with B7H expressed constitutively on naïve B cells but expression extinguishes after antigen engagement and IL-4 cytokine exposure (37). CD40 stimulation of B cells restores B7H expression (37). The fact that ICOS is limited to only B7H stimulation provides possible unique signaling outcomes. CTLA-4, unlike its counterparts, plays a role in cell regulation as opposed to cell activation (36). CTLA-4 is constitutively expressed on Tregs and is inducible on effector cells (38), including potential pathogenic effector cells (39). Study of CTLA-4-mediated tolerance demonstrated interesting outcomes relative to endogenous and exogenous antigens. Using double transgenic mouse models, a more definitive role for CTLA-4 was determined. Mice genetically manipulated to express an ovalbumin peptide directly on islet beta cells, RIPmOVA mice on a BALB/c background, and exposed to OVA-peptide-specific T cells, DO11.TCR-transgenics, only developed diabetes if the cells originated from RAG^−/−^.DO11.TCR.Tg donor mice and if OVA peptide was injected (40). If donor T cells further included a CTLA-4^−/−^ background, then disease occurred independently of injected OVA. Because of CTLA-4’s role as potential tolerance inducer, it was considered an ideal candidate for therapeutic development. A CTLA-4 analog was developed as a therapeutic in T1D, but has had only limited success (41, 42).

Given the potential prominence of CD28 signaling, considered crucial for T cell activation, it was assumed that CD28 would be a useful target for controlling autoimmunity. The assumption being that induced anergy, as suggested by Jenkins and Swartz, would prove therapeutic. This rationale applied to T1D and other autoimmune diseases. CD28 and B7 knockout mice were created but the results were unanticipated. CD28^−/−^ and B7^−/−^ mice developed extensive autoimmunity (36, 43–48). One study showed that in the experimental autoimmune encephalomyelitis model of MS, CD28^−/−^ mice developed very rapid, more severe disease when challenged with disease causing MOG antigen (46). If, however, the CD40–CD154 signaling pathway was blocked, no disease development occurred (46). CTLA-4^−/−^ mice likewise experience extensive, systemic autoimmunity (49, 50). Collectively these data suggested that CD28 plays a more prominent role in regulatory T cell development. In fact, CD28 is required for Treg development (43). Rather than CD28 being the “all-purpose” T cell costimulus it was originally thought to be, its part in autoaggressive T cell stimulation comes in to question.

## Mucin Costimulus

Given that the Immunoglobulin family (CD28/ICOS specifically) knockouts still experienced classic T cell responses; the only viable explanation was “other” costimulatory molecules. A series of T cell potential costimulatory molecules was discovered in relation to Asthma that were defined as T-cell-immunoglobulin-domain/mucin-domain (TIM) proteins. The family is comprised of eight members, two of which occur directly on T cells (51). Only TH2 cells express TIM-1 where it plays a pro-regulatory role while TIM-3 preferentially is expressed on TH1, TC1 cells, and DC (52). TIM-3 engagement results in inhibitory signals that lead to apoptosis (52). Polymorphisms within the TIM family were examined in T1D and no positive correlations were discovered (53). The ligand for TIM-3 is galectin-9 (51). NOD mice that were treated with a plasmid encoding galectin-9 were significantly protected from diabetes (54). In another model, galectin-9 treatment induced aggregation and cell death of TH1 cells, selective loss of IFNγ producing cells and suppression of TH1 autoimmunity (55). Treating mice with anti-TIM-3 resulted in increased fatty streak formation and increased atherosclerotic plaque formation in mice (56). The problem with identifying galectin-9 as the ligand for TIM-3 is that galectins are proteins that non-specifically interact with carbohydrates. Galectin-9 for example interacts with β-galactoside sugars on proteins including CD44 (57) and CD40 (58), both of which are associated with pathogenic T cells. In addition, Galectin-9 interaction with CD40 is independent of TIM-3 (58). A study showed that activation of human T cells is not affected by the presence of galectin-9 nor to antibodies to TIM-3 (59). That result is logical given that galectin-9 can bind to any β-galactoside. Given these findings, many of the TIM-3 studies must be reconsidered in relation to autoimmunity. Galectin-9 for example can interact with any β-galoctoside and, therefore, may impact a large number of signaling pathways not just TIM-3.

## TNF-Receptor-Superfamily Costimulus

A perhaps somewhat surprising subgroup of T cell costimulatory molecules involves members of the TNF-receptor-superfamily (TNFRSF). The initial understanding of TNFRSF costimulation was from the perspective of APCs. The early assumption was based on the determination that TNFRSF ligands or TNF-super family members largely are expressed on activated T cells and the receptors, TNF-receptors I and II, and CD40, etc., were first described on APC. TNFα is a pro-inflammatory cytokine produced by TH1 cells and macrophages (60); it occurs both as a secreted cytokine, the major form, and as a cell surface molecule (61). Both the soluble and transmembrane forms of TNFα interact with TNFR I and II (61). CD154, one of the ligands for CD40, is activation induced on T cells where its expression is temporal (18). CD154 also is expressed on APC (62), astrocytes in the CNS (63), and its major source is platelets (64). Like TNFα, CD154 occurs as both a cell surface molecule and a secreted form (65–67). Soluble CD154 is significantly increased in serum of T1D (68), and other autoimmune diseases; it may behave as a highly pro-inflammatory cytokine.

Members of the TNFRSF that act as T cell costimulatory molecules include 4-1BB and OX40. Both OX40 and 4-1BB are activation induced and promote cell survival, potential T cell memory formation, and cytokine production (69–71). In addition to expression on effector T cells, OX40 was detected on CD25^+^, potential Tregs in T1D patients (72). 4-1BB performs similar function on T cells (73, 74). 4-1BB mapped to the *Idd9.3* locus in NOD mouse studies, and reportedly increases IL-2 production and improves CD3 stimulated-activation-outcomes (75–77). These data suggest that OX40 and 4-1BB are more directed toward regulatory outcomes. In that same vein, another TNFRSF member is glucocorticoid-induced-TNF-receptor-protein, GITR known as TNFRSF18. GITR is predominately associated with Tregs (38). Like OX40 and 4-1BB, GITR increases IL-2 production, and improves CD3 activation, developing the MAPK signaling cascade (38, 78). Tregs have been discriminated into innate, those that arise during thymic development (79, 80), and induced, Tregs that are created in the periphery often after exposure to IL-10, GITR expression associates with induced Tregs (38, 79–82).

## CD40 (TNSFR5)

Unlike the other TNF-receptor costimulatory molecules on T cells, CD40 acts in a predominant pro-inflammatory manner (18, 27, 31, 58, 83–99). CD40 expression was first described on B cells, and when associated with IL-4, CD40 signals induce antibody class switching. While this action could be involved in autoantibody generation, such function has not been described in T1D or other autoimmune diseases. Like other TNFRSF members, CD40 signals ablate cell death and promote cell survival in B cells, performing similar function in T cells (22, 100). A major problem in understanding the scope of CD40-mediated inflammation has been a gross underestimation of CD40 expression. As studies of CD40 evolved, its expression was identified in numerous cell types. CD40 is expressed on all professional APC, B cells, but also DCs and macrophages. On DCs, it plays a central role in T cell licensing. CD40 engagement on DC switches the DC’s interactions with T cells (101). DCs that are high CD40 expressers promote TH1 cell development while CD40-low or CD40-negative DCs favor Treg development (102). CD40 induces iNOS in macrophages (103), thus contributing to the innate immune arm and it induces pro-inflammatory cytokines, including TNFα, IL-1α, IL-1β, and IL-6 (17, 18, 104). CD40 expression has been described on endothelial cells (105); neural cells (106); and surprisingly on islet β cells (107–109). On each of those cell types, CD40 engagement leads to pro-inflammatory cytokine production.

While initially unexpected, CD40 expression occurs on T cells, including CD4^+^ and CD8^+^ cells (20–23, 26–28, 31, 39, 58, 100, 110–113). Like OX40 and 4-1BB, CD40 on CD8^+^ cells is associated with memory cell generation (114). On CD4^+^ cells, CD40 has been reported on naïve, effector, central, and effector memory cells (29–31), in both murine and human studies. CD40 engagement works independently of CD28 or other costimulatory molecules, inducing predominantly TH1 phenotype cytokines including TNFα and IL-6 (29), as well as GM-CSF and IL-1β (31). CD40 costimulus also induces the TH17 phenotype cytokines IL-17 and IL-21. Interestingly, the TH1 and TH17 cytokines express concomitantly in TH40 cells after CD40 engagement. Because TH40 cells produce both TH1 and TH17 cytokines, post CD40-mediated costimulus these helper cells do not fit the paradigm of either TH1 or TH17 cells, and thus have been termed TH40 cells (20–22, 27, 28, 39, 100, 112, 113).

## TH40 Cells: CD40 Serves as a Biomarker for Autoaggressive T Cells

When isolated from diabetic or pre-diabetic NOD mice TH40 cells transfer diabetes readily and without any manipulations; thus CD40 constitutes a diabetogenic T cell biomarker (20–22, 26–28, 100). A panel of highly pathogenic, autoaggressive T cell clones, including the well described BDC2.5 and BDC6.9 clones, express CD40 (20, 21, 28). Non-diabetogenic T cell clones including BDC2.4, isolated from the same NOD spleen as BDC2.5 cells, do not express CD40 (28). Primary TH40 cells increase to significantly greater percentages and cell numbers during autoimmunity (20–22, 26, 27, 100). However, like Tregs, some CD40-expressing CD4 cells arise in the thymus (39). In NOD mice that develop spontaneous diabetes, substantial thymic increases in numbers of CD40^+^ thymocytes were observed (111). Likewise, in a double transgenic, neo-self-antigen model, DO11.RIPmOVA mice, where TCR.Tg T cells that are specific for OVA encounter OVA on thymic medullary epithelial cells, thymic CD40 expressing CD4^+^ cells were significantly expanded in number (39). The percentage of developing TH40 thymocytes in NOD mice was identical to that of DO11.RIPmOVA mice, suggesting that auto-antigen drives the expansion of TH40 cells in the thymus. During T1D, the percentage of TH40 cells expands proportionately with increasing insulitis over time in NOD mice (29). In fact, TH40 cells proved to be diagnostic for T1D. In female NOD mice, 80% develop T1D by 18–22 weeks of age, while only 20–50% of male mice develop disease. Observations reveal that diabetic male NOD had peripheral TH40 cell numbers equivalent to that of diabetic female mice (Wagner Lab observations). Likewise, in NOD female mice that did not develop diabetes, TH40 cell numbers remain at numbers found in non-autoimmune mice. These observations suggest that breach of tolerance involves TH40 cell number expansions.

Primary TH40 cells isolated directly from the pancreatic lymph nodes or spleens of pre-diabetic and diabetic NOD mice transferred progressive insulitis and diabetes to NOD.*scid* recipients (21, 28). CD40^−^ T cells did not transfer disease, even after removal of Tregs and additional *ex vivo* activation (20–22, 28). T cell CD40 expression is long-lived unlike classic activation molecules, i.e., CD69, CD25, or CD154. Furthermore, classic TCR-mediated activation of naïve CD40^−^ T cells does not induce CD40 expression. Interestingly, in murine studies, TH40 cells are less susceptible to Treg suppression than non-CD40 expressing T cells (22). TH40 cells are able to express CTLA-4, one of the immunoglobulin family, pro-regulatory molecules. Using the neo-self-antigen model of T1D, TH40 (DO11 TCR^+^) cells isolated from diabetic mice did not express CTLA-4, while the vast majority of CD40^−^ T cells (also DO11 TCR^+^) were CTLA-4^+^ (39). CTLA-4 is activation induced, requiring TCR engagement (38); therefore CTLA-4 expression may be regulated by CD40-mediated signals as was demonstrated (39). Observations further show that in diabetes prone NOD mice expression of CTLA-4 is deficient (39).

## Battle of the Costimulatory Molecules

**Figure 1:**
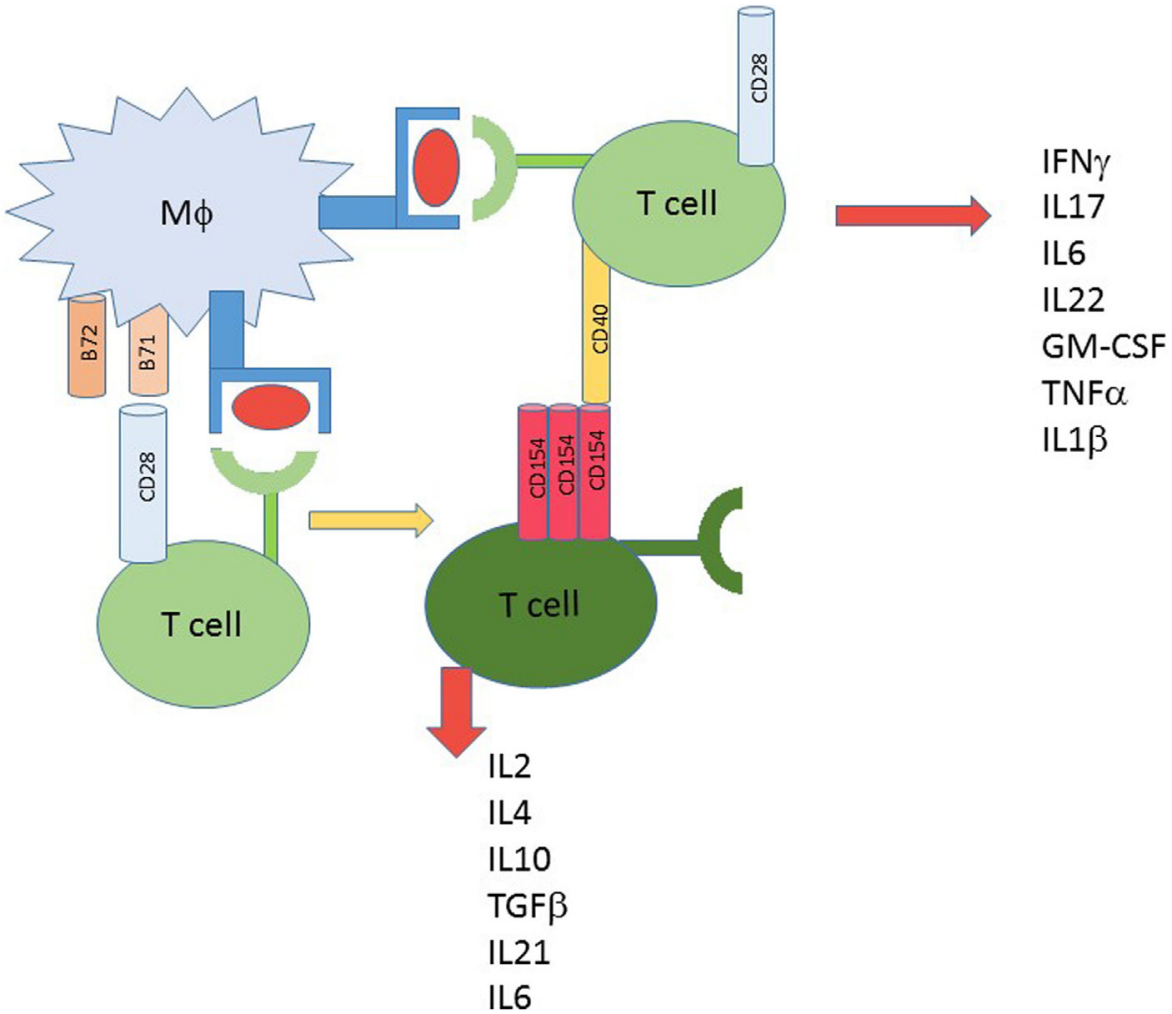
T cell activation alternative methods. Classic 2 signal activation involves T cell interaction with antigen presenting cell. Signal one is antigen dependent and delivered to the T cell receptor by MHC/HLA and antigen. The costimulus can include CD28 interacting with B71 or B72; that outcome creates activation leading to cytokine production. When a source of CD154 is available, including from the newly activated T cells, other T cells receive a different second signal costimulus to produce inflammatory type cytokines. CD28 signals favor more regulatory type cytokines.

Clearly, T cells express an array of costimulatory molecules and while each can contribute to activation, the reality is that different costimulators drive the T cell in different directions. CD28, for many years, was considered the central T cell costimulatory molecule. Virtually all *in vitro* T cell stimulation/activation protocols utilized CD28 costimulation. What became a surprise was that CD28 interaction with B71 or B72 drives a more regulatory phenotype leading to production of IL-4, IL-10 and especially IL-2 (Figure 1). The role of IL-2 will be discussed further below. One reason that CD28^−^ only costimulation became universally accepted as pro-inflammatory is that the coincident response of CD40–CD154 interaction was overlooked. When CD40 is engaged on T cells, independently of CD28 (29–31, 110) or in combination with CD28 (30, 31, 110), a TH1/TH17 pro-inflammatory phenotype results (Figure 1). When T cell activation occurs, signals through the synapse lead to induction of CD154 expression (115). Therefore, in the microenvironment a source for CD40–CD154 interaction in addition to CD28, costimulation is developed. In T1D compared to controls, CD40-bearing T cells, TH40 cells in particular, are over represented (20–22, 24, 27–29, 31, 39, 90, 100, 110–112, 116). The overlooked issue is T cell–T cell interactions (Figure 1). An activated T cell is an abundant source of CD154 and can, therefore, interact with CD40 on APC, within the immune synapse; but it also can interact with CD40 on TH40 cells. That interaction leads to inflammatory cytokine production (23, 29–31, 39, 58, 110–112), as represented in Figure 1. Importantly, the CD40 signal can override the CD28-mediated signal (29, 30), to drive inflammation.

## The Unintentionally Misunderstood Role of IL-2

IL-2 discovery began from an accidental, but important happenstance involving the kidney bean extract, phytohemagglutinin (PHA). When cells in culture were treated with the extract, there was lymphocyte cellular expansion (117). The expansion was temporary, however. Over time, the substances induced by PHA that caused leukocytes to proliferate were identified as interleukins and the interleukin associated with T cell expansion specifically became known as IL-2. After this discovery, the addition of IL-2 to all T cell cultures was considered essential. To better understand T cells and IL-2 responses specifically promoting autoimmunity, IL-2 knockout mice were generated. The *a priori* hypothesis was that autoimmune disease would be completely negated, since theoretically IL-2 would be the cornerstone T cell survival cytokine. Surprisingly, IL-2^−/−^ mice demonstrated that IL-2 was not the crucial cytokine for T cell development that it was thought to be (118, 119). Effector T cells develop normally in the thymus in the absence of IL-2 (120) and in fact, autoimmunity is abundant and spontaneous in those mice (121). IL-2 is, however, required for Treg development and homeostasis (120). Further study demonstrates that IL-2 promotes Treg development but does not promote pathogenic effector cell development (120). This observation creates unique problems for the vast array of T cell *in vitro* experiments where IL-2 has been added as the T cell costimulus for pathogenic effector cells. Those studies require re-visiting given that IL-2 promotes regulation.

## Alternate Mechanisms for Effector T Cell Regulation: TH40 Cells Expressing FOXP3

**Figure 2:**
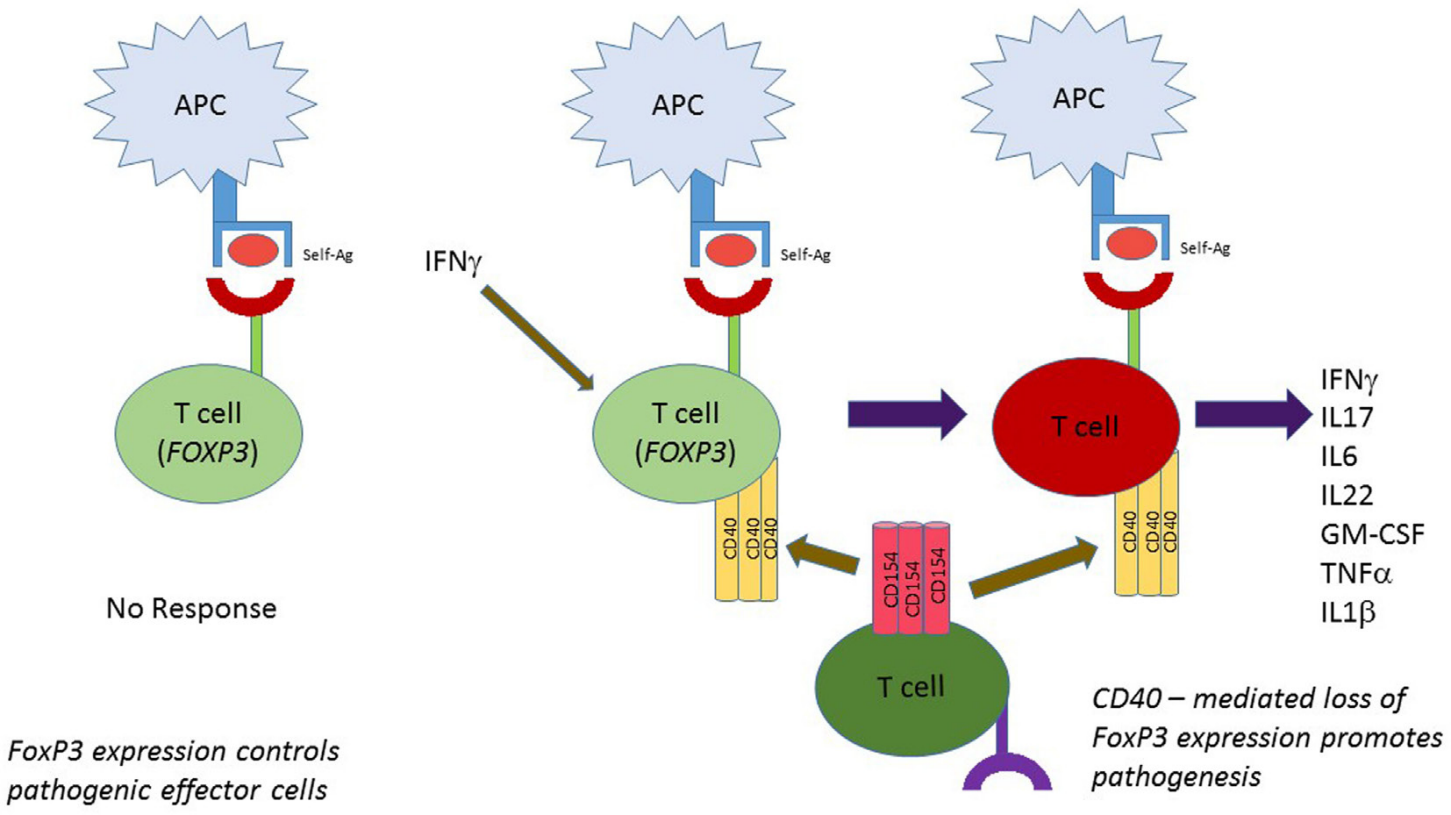
FOXP3 as a static T cell regulator independent of Treg status. Cells other than classic Tregs express FOXP3. In the BDC2.5 T cell receptor transgenic mouse model effector, T cells express FOXP3 but in the inflammatory environment where IFNγ, etc., is produced, CD40 is induced on those cells. CD40 interaction with CD154 leads to loss of FOXP3 expression and full effector status.

The BDC2.5 T cell clone rapidly and efficiently transfers diabetes to NOD.scid recipient mice (20, 28, 122). When the TCR transgenic (TCR.Tg) version of BDC2.5 was created, only about 22% of mice became diabetic in the time frame considered (123). This outcome was surprising, given the strong diabetogenicity of the BDC2.5 clone. Longitudinal studies revealed a different outcome, however. If BDC2.5.TCR transgenic mice were followed for 45 weeks, disease incidence achieved 100% (111). That finding suggests that eventual breach of tolerance occurs. TH40 cells expand rapidly in the BDC2.5.TCR.Tg mouse model, but disease kinetics are much slower than in classic NOD mice (111). The unexpected finding was that TH40 cells at younger ages contained high levels of FOXP3 (111). In this circumstance TH40 cells were not Tregs; the BDC2.5.TCR.Tg mice maintained classically defined Tregs, CD4^+^CD25^hi^FOXP3^+^. Tregs but not TH40-FOXP3^+^ cells performed regulatory functions (111). The disease defining parameter was loss of FOXP3 in TH40 cells. TH40 cells isolated from young animals had high FOXP3 levels and could not transfer diabetes; TH40 cells that rapidly and efficiently transferred diabetes were FOXP3 negative regardless of the age of the donor (111). Not only were TH40 cells disease instrumental, but CD40 itself proved to be a disease master switch. BDC2.5.TCR.Tg mice bred onto the CD40 knockout background did not develop diabetes at any age (111) and equally impressive, those mice did not exhibit insulitis (29). In addition, BDC2.5 T cells isolated from the CD40 KO mice maintained high levels of FOXP3 even after 45 weeks. These findings indicate that when CD40 levels are sufficiently controlled, effector cells are able to express FOXP3. Furthermore, that expression is independent of Treg status. If systemic CD40 levels and CD154 levels are substantially elevated, just as occurs in T1D, then effector cells loose FOXP3 to become pathogenic effector cells (Figure 2).

## Conclusion

Failure of central tolerance cannot be excluded as an autoimmune mechanism. Numerous studies demonstrate that negative selection failure gives rise to peripheral pathogenic T cells. However, the overlooked mechanism of TCR revision also must be considered. This process that increases overall T cell repertoire to pathogens likewise increases risk for autoimmunity. If appropriate selective pressures are not applied in the periphery following TCR revision, then autoaggressive T cells necessarily will arise. For autoimmune disease to commence, additional criteria must be met, including perhaps HLA haplotype, CTLA-4, FOXP3 or other mechanisms of tolerance failure, etc. Within these bounds the role of costimulation is becoming evident as contributory for autoimmunity. The classic CD28 family has been thought essential for driving T cell expansions, but overlooked costimulation, CD40 in particular, is emerging as diabetogenic. TH40 cells become prominent in the NOD mouse model of T1D as well as in human T1D (20, 21, 26, 27). These cells have clearly proven to be important disease drivers. Of significance is that the CD40 molecule itself acts as a highly pro-inflammatory stimulator (29, 30). When T cell CD40 is directly engaged, T cells produce TH1 and TH2 cytokines; but when CD28 is engaged, T cells produce TH2/Treg cytokines (29, 30). Another concern is that classic tolerance mechanisms driven by Tregs or by tolerance inducing molecules, CTLA-4 and FoxP3 for example, work much less efficiently on TH40 cells derived from autoimmune backgrounds (39, 111). These findings include tolerance dysfunction in human T1D (26, 27). Full understanding of disease mechanisms leading to breach of tolerance in T1D and other autoimmune diseases is a complicated process. No one mechanism alone is causative. Nonetheless a clearer picture of those processes involving TCR development and T cell costimulation is beginning to emerge.

## Author Contributions

DW conceived and wrote the manuscript.

## Conflict of Interest Statement

DW is Chief Scientific Officer of Op-T, LLC.
